# The effects of serial assessment using the trail making test in elite rugby league players

**DOI:** 10.1093/arclin/acag082

**Published:** 2026-10-15

**Authors:** Cassandra Richardson, Stephen Atkins, Howard Hurst, Ian Bentley, Jonathan Kenneth Sinclair

**Affiliations:** School of Psychology and Social Sciences, University of Winchester, Winchester SO22 4NR, UK; School of Health & Society, University of Salford, Salford M5 4WT, UK; School of Health, Social Work and Sport, University of Lancashire, Preston PR1 2HE, UK; School of Health, Social Work and Sport, University of Lancashire, Preston PR1 2HE, UK; Wigan Warriors RLFC, Wigan WN5 0UH, UK; School of Health, Social Work and Sport, University of Lancashire, Preston PR1 2HE, UK

**Keywords:** assessment, attention, executive functions, head injury, traumatic brain injury, perception/spatial processing, practice effects/reliable change

## Abstract

**Objective:**

The aim of the study was to map trajectories of inter- and intra-individual cognitive performance in non-injured elite rugby players to further understand the effects of serial cognitive assessment in athletes at risk of concussion.

**Method:**

The Trail Making Test (TMT) was administered to male elite rugby league players (*N* = 38) at three time-points pre-season and on average 6.8 times per month during a playing season. Three players were excluded from the analyses due to concussion or suspected concussion. The data were analyzed using multilevel models including fixed and random effects of time to assess within- and between-individual variability.

**Results:**

Time was the greatest predictor of both TMT-A and TMT-B scores; test performance improved until it reached a plateau and then remained stable. The reduction of within-individual variance indicated that test repetition contributed to improvements in TMT-A and TMT-B performance within each player. Importantly, performance was significantly different between players at first testing and over the course of a playing season which indicated performance heterogeneity within one cohort of elite rugby league players.

**Conclusions:**

The current study has contributed to further understanding pre-injury cognitive performance in elite rugby players and indicated the need for future studies to implement strategies for reducing practice effects. Most importantly, these findings are relevant when assessing cognitive functioning in athletes at risk of concussion. This study emphasized the need to account for variability in performance pre-injury, the assessment of potential performance confounds and recommends the application of methods to reduce practice effects.

The Trail Making Test (TMT) is comprised of two parts, A and B (TMT-A; TMT-B; Reitan, 1958) and is a common component of neuropsychological test batteries that assess cognitive functioning after traumatic brain injury (TBI; e.g., Brett et al., 2021). The TMT measures visual searching and visuospatial sequencing, processing speed and cognitive flexibility, and is sensitive to TBI (Lezak et al., 2012). Sport-related concussion (SRC) is defined as a TBI (Patricios et al., 2023) and the term sports-related mild traumatic brain injury (SR-mTBI) is increasingly being used (e.g., Haste et al., 2025). A recent narrative review found that attention, reaction time (RT) and motor speed are most affected by SR-mTBI, with attentional impairments taking the longest to resolve post-injury (Ahn et al., 2026). There has been a call to improve the management of cognitive deficits following SR-mTBI (Hallock et al., 2023). However, there are well-documented challenges with using neuropsychological tests to monitor cognitive function over time, namely, practice effects and debates about baseline assessment and reliable change calculations (e.g., Attix et al., 2009; Calamia et al., 2012; Echemendia et al., 2013; Heilbronner et al., 2010; Iverson & Schatz, 2015). The TMT is highly recommended as a core element of SR-mTBI assessment (Broglio et al., 2018). Mapping typical performance trajectories for athletes at risk of concussion (e.g., rugby league players) is essential for interpreting their test scores in a clinical setting and contributes to discussions about baseline assessment and reliable change indices.

Practice effects are improvements in test performance with repeated administration, influenced by factors such as test familiarity, motivation and regression to the mean rather than *actual* changes in cognitive ability (Heilbronner et al., 2010). Acceptable test–retest coefficients have been reported (TMT-A: *r* = 0.70–0.87; TMT-B: *r* = 0.70–0.89; Amodio et al., 2002; Dikmen et al., 1999; Levine et al., 2004), indicating that the TMT is a reliable measure of cognitive functioning in healthy adults. However, reliability coefficients do not inform on practice effects. Practice effects are found for the TMT (e.g., Bartels et al., 2010; Dikmen et al., 1999; Levine et al., 2004) indicating that performance significantly improves at retest, but effect sizes are highly variable (0.08–0.75; Bartels et al., 2010; Dikmen et al., 1999; Levine et al., 2004). The greatest improvements in TMT performance were found with high frequency testing (5 testing time-points) over a 3-month period after which performance plateaued and did not significantly change from 3 to 12 months (Bartels et al., 2010). A seminal meta-analysis of practice effects found TMT-A and TMT-B scores increased by ~20% of the test standard deviation (β = 0.19 and 0.21, TMT-A and TMT-B, respectively) at retest (Calamia et al., 2012). Furthermore, TMT performance gains with test repetition were found to decrease with increasing age and with length of test–retest interval (Calamia et al., 2012). The data provided by Calamia and coworkers (2012) are invaluable for clinical practice and research, as practice effects for different ages (40+ years), lengthy time intervals (≥1 year) and different clinical populations can be calculated. However, the application to younger adults and testing intervals of <1 year is limited due to the reference category (healthy 40-year-old evaluated at 1-year interval). More research is warranted in younger, non-clinical populations with multiple testing intervals to identify practice effects and patterns of inter- and intra-individual performance change over time.

The TMT has been included in neuropsychological test batteries to assess cognitive function in athletes at risk of concussion, facilitating evaluation of how individual tests and/or entire batteries contribute to the screening and monitoring of concussions (e.g., Collie et al., 2006; Collins et al., 1999; Fuller et al., 2021; Julien et al., 2023; McCrea et al., 2005; Merritt et al., 2017). There is evidence that athletes outperform controls in a variety of cognitive domains (e.g., Gu et al., 2019; Logan et al., 2022). Improved performance in specific types of athletes (e.g., high intensity interval training team trained athletes and elite athletes) significantly contributed to the cognitive differences between athletes and non-athletic controls (Logan et al., 2022). TMT practice effects have also been found in contact sport athletes (Collie et al., 2006; Julien et al., 2023). In addition, poor agreement (*ρc* = 0.30) in TMT-B scores (T1–T2) in elite rugby players using an abridged digital version of the TMT-B, indicated highly variable performance within players (Fuller et al., 2021). These findings call into question the reliability of baseline assessment as an index of pre-injury performance. In support, only 5.8% of college athletes would have diagnostically benefitted from baseline assessment over published normative values (Pandey et al., 2025). There is also evidence that baseline assessment includes high rates of failure (Abeare et al., 2018) and deliberate poor performance is a concern (Echemendia et al., 2013). However, experimental manipulation of “sandbagging” indicated only 10.7% of college athletes can fake performance without detection by performance indicators, such as published population norms (Erdal, 2012). These findings contribute to an argument for the value and importance of normative data over baseline assessment. Of note, normative data are used to calculate a minimal clinically important difference (MCID), a commonly utilized method to calculate reliable change in randomized controlled trials and by clinicians (Hinton-Bayre, 2010; Wright et al., 2012).

When focusing on post-injury TMT scores and identification of injury-related change, different patterns of TMT performance have been reported in athletes after sustaining a concussion: no significant difference in TMT-B scores between concussed and non-concussed rugby players (Fuller et al., 2021); a lack of practice effects in the TMT-B in Australian Rules footballers with symptomatic concussion (Collie et al., 2006); an improvement in TMT-A and B performance post-concussion in rugby players (Julien et al., 2023); and better TMT-B performance in footballers with asymptomatic concussion (T1–T2; Collie et al., 2006). These findings are not easily reconciled due to variability in TMT version used (e.g., pencil and paper: Collie et al., 2006; Julien et al., 2023; abridged digital: Fuller et al., 2021), study design and injury-to-measurement time. Whilst the use of the TMT as a screening measure may not be supported (Fuller et al., 2021), there is not a single gold standard measure, and multimodal assessments demonstrate the best accuracy for diagnosing SR-mTBI (Haste et al., 2025). These conclusions do not preclude the use of the TMT to monitor cognitive functioning in athletes at risk of concussion or post-concussion. Rather, additional research is warranted to investigate trajectories of inter- and intra-individual cognitive performance in specific non-injured athletic groups to facilitate understanding of practice effects with serial assessment. Such data contribute to discussions about reliable change calculations as inter- and intra-individual variability are evident in the TMT (see Fuller et al., 2021; Julien et al., 2023) and normative data are specific to the version of the test used, and possibly, to the type of athlete (cf. Logan et al., 2022).

In order to address the specific need to understand inter- and intra-individual variability with serial assessment as discussed above, the aim of the current study was to examine TMT performance in elite rugby league players across one playing season. This study was an extension of our previous study which collapsed TMT performance across baseline assessment data points to compare with pre-season TMT performance (Richardson et al., 2017). There is evidence that multiple trials of a test are needed to reach a ceiling (see Gunasekaran et al., 2020) and that there is significant within and between-person variability in neuropsychological test performance in rugby players (e.g., Fuller et al., 2021; Julien et al., 2023). As such, this was an exploratory study due to the paucity of studies investigating high frequency serial assessment of cognitive functioning across a playing season in elite rugby players using the TMT. Therefore, the research question was: how does performance on the TMT-A and TMT-B change over the course of a playing season in elite rugby league players without concussion?

## Method

### Participants

Thirty-eight male elite rugby league players (*M* age 25.1 years, *SD* = 4.5) contracted to an English professional rugby club taking part in the Super League participated in the study. Three players were excluded from the analyses due to concussion or suspected concussion. The concussion incidence rate was 1.91 per 100 person-months, 95% CI [0.38, 3.44]. Incidence rate was calculated by dividing the number of concussions in the observation period by the total person-time of observation whilst at risk of concussion. Ethical approval for the study was obtained from the University of Lancashire review board and all participants provided written informed consent.

### Materials

TMT-A required participants to draw a line sequentially from circled numbers 1–25 without taking the pen/pencil off the paper (total distance = 111.5 cm; a shorter version was used to facilitate comparison with abridged digital versions of the TMT). The stimuli in the TMT-B were circled numbers and letters and participants were required to draw a line from a number to a letter in alternating order (1-A-2-B-3-C etc.; total distance 127.5 cm). Sample A (circled 1–8) and B (circled 1-A-2-B-3-C-4-D) were administered as task familiarization prior to each administration of each part of the TMT. Scoring was time taken in seconds to complete each part, manually measured using a digital stopwatch, with better performance indicated by lower scores. The TMT ratio was also calculated (TMT-B/TMT-A). The TMT B/A ratio indexes attentional control processes (Arbuthnott & Frank, 2000) and controls for factors linked to intra-individual variability (e.g., age and education; Drane et al., 2002; Lamberty et al., 1994).

### Procedure

The TMT was administered to available participants on testing days; not all participants completed the TMT at each testing time point. Testing was arranged on a weekly basis on days and times convenient to the club. All data were collected in an unobtrusive location at the rugby club on training days and before some matches. To account for potential data collection bias, data were collected by trained researchers, rather than the research team, using the standardized instructions provided in Spreen and Strauss (1998) with an emphasis on speed. The TMT-A was always administered immediately before the TMT-B. Data were collected at 57 time-points during the study; time 1–3 (T1–T3) were collected prior to the start of the season within a 9-day period. During the season, data were collected on average 6.8 times per month.

### Statistical analysis

The data were analyzed using multilevel models as these longitudinal data have a two-level hierarchal structure as multiple observations (level 1) are nested within individuals (level 2). The MIXED procedure in SPSS v.30 was used with the Restricted Maximum Likelihood (REML) estimation method. REML is recommended for small samples (*N* < 50), produces less biased estimates of between-individual variance parameters and more reliable standard errors (Snijders & Bosker, 2012). Curve fitting was conducted using GraphPad Prism v.9 to model change over time and found that an exponential decay model provided a good fit for the data *R^2^* = 0.48 and 0.57, TMT-A and TMT-B, respectively. The exponential decay models indicated that there was a rapid improvement in performance of each test, followed by minimal improvement, until performance reached a ceiling and remained stable.

The distributions of the TMT-A and TMT-B scores were positively skewed. Outliers were investigated due to the potential to influence variance estimates in multilevel models. Outliers were identified as influential time series additive outliers rather than as single construct or error outliers. As scores represent behavioural performance, distraction during testing, or an “off” day could explain outlier scores. Following the recommendations of Aguinis and coworkers (2013), influential outliers were identified using Cook’s *D* with the cut-off of 4/*n*. Each outlier was investigated and removed if it was noticeably greater than other scores for that time-point and identified as an outlier on a boxplot. After outliers were removed, mean (*SD*) TMT scores and B/A ratios were calculated for each time point (see Table 1). The 25th percentile was used to identify below average performance (see Table 1).

**Table 1:** Sample size, mean (SD) scores in seconds, number and percentage of participants in the 25th percentile for the TMT-A and TMT-B, and the mean (SD) of the TMT ratio for each testing time point

Time Point	TMT-A			TMT-B			Ratio (B/A)
	(*n*)	Mean (*SD*)	25^th^ Percentile Number (%)	(*n*)	Mean (*SD*)	25^th^ Percentile Number (%)	Mean (*SD*)
1	32	16.5 (3.3)	9 (28.1)	32	40.4 (13.3)	8 (25.0)	2.49 (0.87)
2	29	14.1 (2.8)	7 (24.1)	31	34.4 (12)	7 (22.6)	2.38 (0.78)
3	31	14.3 (3.0)	9 (29.0)	30	30.4 (8.4)	9 (30.0)	2.20 (0.62)
4	23	12.8 (2.5)	6 (26.1)	23	27.7 (7.3)	7 (30.4)	2.12 (0.56)
5	27	12.8 (2.7)	5 (18.5)	28	27.8 (8.3)	7 (25.0)	2.17 (0.68)
6	24	12.2 (2.8)	7 (29.2)	24	24.9 (8.9)	6 (25.0)	2.08 (0.72)
7	24	11.7 (2.4)	6 (25.0)	24	22.3 (6.5)	4 (16.7)	1.93 (0.44)
8	21	11.8 (2.6)	5 (23.8)	21	22.4 (7.7)	4 (19.0)	1.86 (0.46)
9	20	11.0 (1.9)	6 (30.0)	18	20.5 (4.8)	7 (38.9)	1.89 (0.38)
10	19	9.9 (1.5)	5 (26.3)	20	18.7 (5.3)	4 (20.0)	1.86 (0.45)
11	28	10.8 (1.9)	6 (21.4)	30	20.8 (6.8)	7 (23.3)	1.86 (0.47)
12	21	10.6 (2.2)	7 (33.3)	21	20.8 (7.7)	6 (28.6)	2.00 (0.73)
13	22	10.0 (1.9)	4 (18.2)	23	19.4 (7.7)	7 (30.4)	1.96 (0.64)
14	26	10.1 (2.3)	8 (30.8)	25	18.1 (6.2)	7 (28.0)	1.79 (0.47)
15	23	9.4 (1.4)	5 (21.7)	22	16.2 (5.6)	4 (18.2)	1.68 (0.42)
16	20	9.7 (1.7)	4 (20.0)	19	18.1 (5.0)	5 (26.3)	1.93 (0.54)
17	19	9.1 (1.6)	5 (26.3)	19	16.4 (5.6)	4 (21.1)	1.81 (0.48)
18	22	10.4 (1.8)	5 (22.7)	22	16.0 (4.0)	6 (27.3)	1.54 (0.29)
19	21	9.4 (2.2)	6 (28.6)	21	17.1 (6.3)	5 (23.8)	1.82 (0.46)
20	15	9.0 (2.2)	4 (26.7)	16	16.5 (8.0)	4 (25.0)	1.76 (0.79)
21	20	9.8 (2.5)	5 (25.0)	19	15.7 (4.5)	3 (15.8)	1.65 (0.48)
22	19	9.0 (1.7)	4 (21.1)	16	12.2 (2.7)	4 (25.0)	1.41 (0.23)
23	19	8.9 (1.6)	6 (31.6)	18	14.6 (3.3)	5 (27.8)	1.66 (0.20)
24	17	8.5 (1.3)	6 (35.3)	17	13.9 (3.4)	6 (35.3)	1.64 (0.31)
25	15	7.5 (1.7)	5 (33.3)	14	11.8 (3.2)	2 (14.3)	1.70 (0.71)
26	22	9.6 (1.7)	6 (27.3)	22	14.0 (2.9)	5 (22.7)	1.47 (0.27)
27	21	9.6 (2.1)	5 (23.8)	21	14.3 (2.9)	7 (33.3)	1.52 (0.30)
28	19	8.7 (1.4)	4 (21.1)	18	13.1 (2.1)	4 (22.2)	1.53 (0.27)
29	18	8.4 (1.3)	4 (22.2)	19	13.4 (2.6)	5 (26.3)	1.60 (0.27)
30	22	9.1 (1.9)	8 (36.4)	21	13.3 (2.7)	4 (19.0)	1.52 (0.34)
31	21	9.8 (1.8)	5 (23.8)	20	13.5 (2.6)	5 (25.0)	1.40 (0.32)
32	22	9.0 (1.7)	4 (18.2)	23	13.4 (2.8)	6 (26.1)	1.50 (0.34)
33	17	8.4 (1.1)	3 (17.6)	18	12.7 (2.7)	3 (16.7)	1.51 (0.30)
34	17	8.8 (1.5)	5 (29.4)	16	13.2 (2.8)	4 (25.0)	1.54 (0.32)
35	21	8.5 (1.2)	5 (23.8)	21	13.8 (3.6)	5 (23.8)	1.64 (0.44)
36	23	8.9 (1.6)	3 (13.0)	24	14.5 (3.9)	6 (25.0)	1.63 (0.52)
37	23	8.8 (1.7)	5 (21.7)	23	14.0 (3.0)	5 (21.7)	1.65 (0.50)
38	20	8.3 (1.1)	5 (25.0)	21	13.5 (3.6)	5 (23.8)	1.61 (0.41)
39	22	8.7 (1.8)	6 (27.3)	21	13.6 (2.8)	4 (19.0)	1.60 (0.36)
40	23	8.9 (1.5)	4 (17.4)	23	13.2 (3.1)	5 (21.7)	1.49 (0.30)
41	21	9.1 (2.1)	6 (28.6)	20	13.0 (2.2)	3 (15.0)	1.50 (0.35)
42	20	8.4 (1.4)	5 (25.0)	19	13.3 (2.4)	3 (15.8)	1.60 (0.35)
43	19	8.6 (1.3)	5 (26.3)	19	13.6 (2.9)	6 (31.6)	1.60 (0.33)
44	24	8.8 (1.3)	5 (20.8)	24	14.0 (3.9)	7 (29.2)	1.59 (0.40)
45	22	8.2 (1.2)	5 (22.7)	22	12.8 (2.3)	4 (18.2)	1.56 (0.39)
46	21	8.5 (1.4)	5 (23.8)	21	12.9 (2.4)	4 (19.0)	1.56 (0.30)
47	21	8.2 (1.3)	5 (23.8)	21	14.3 (3.5)	6 (28.6)	1.74 (0.35)
48	26	8.7 (1.5)	6 (23.1)	25	14.3 (2.5)	6 (24.0)	1.69 (0.34)
49	25	8.3 (1.3)	7 (28.0)	24	14.5 (2.8)	5 (20.8)	1.79 (0.41)
50	26	8.6 (1.3)	7 (26.9)	27	14.5 (2.8)	6 (22.2)	1.68 (0.35)
51	16	8.2 (1.8)	4 (25.0)	15	14.0 (3.1)	2 (13.3)	1.76 (0.25)
52	22	8.7 (1.1)	5 (22.7)	23	15.0 (2.5)	6 (26.1)	1.74 (0.30)
53	22	8.7 (1.1)	6 (27.3)	22	15.1 (2.5)	6 (27.3)	1.76 (0.30)
54	22	8.7 (1.4)	7 (31.8)	22	15.2 (2.5)	6 (27.3)	1.79 (0.38)
55	23	8.7 (1.5)	5 (21.7)	21	14.9 (1.8)	5 (23.8)	1.78 (0.37)
56	22	9.0 (1.6)	6 (27.3)	22	15.3 (3.3)	4 (18.2)	1.72 (0.27)
57	23	8.7 (1.6)	7 (30.4)	22	14.7 (2.6)	5 (22.7)	1.75 (0.32)

Due to the pragmatic nature of the data collection, there were missing data (36.5% in both TMT-A and TMT-B). After outlier removal, missing data increased to 37.7% for the TMT-A and 38.1% for the TMT-B. A visual inspection found that the missing data varied across time-points and between participants, and Little’s MCAR test indicated that the data were missing completely at random (MCAR) as *P* > 0.05 for both TMT-A and TMT-B. Although reliable imputation methods are available for handling missing data (see Jakobsen et al., 2017), the substantial amount of missing data precluded imputation. One of the advantages of using multilevel models to analyze longitudinal data is that these models do not assume complete sets of observations (Hox et al., 2018); each data point contributes to the estimation of both within-individual and between-individual variance (Snijders & Bosker, 2012).

Initially, an unconditional model (Model 1) was fitted to the TMT-A and TMT-B data, separately, to provide estimates for within and between-individual variances and to calculate the intra-class correlation coefficient type 1 [ICC(1)] (see Supplementary Material for statistical analysis of the TMT B/A ratio data). The −2 log-likelihood (−2LL) statistic provided a baseline of the model fit against which subsequent models were compared. The exponential decay model of time was added as a fixed effect in an unconditional fixed growth model (Model 2). In Model 3, the exponential decay model was added as a random effect to examine between-individual differences in within-individual change over time. Testing time-point (pre-season, match day, within 1 week of a match day or more than a week from match day) was added to Model 4 as a fixed effect to examine if different testing time-points influenced TMT-A and TMT-B scores. Model 5 included the exponential decay model of time^*^testing time-point interaction term to assess whether testing time-point influenced change in TMT-A and TMT-B scores over time. Within-person variance was modelled in Model 6 to account for the serial correlation of scores between adjacent time-points and account for the non-independence of TMT test scores.

## Results

### TMT-A

The initial unconditional model (Model 1) found that the variance term for the intercept was significantly greater than zero (see Table 2), which indicated that there was significant variability in the intercepts between the players. The ICC(1) was calculated: 3.12/(5.46 + 3.12) = 0.36 and indicated that 36% of the variance in TMT-A scores was attributable to differences between players. The inclusion of the fixed effect of time in Model 2 improved the model fit. However, it was not possible to statistically test this decrease as REML was used in analyses. As time was modelled as a decrease followed by a plateau, the coefficient indicated that there was a positive relationship between time and TMT-A scores; therefore, TMT-A scores decreased over time followed by a ceiling (see Table 1 and Fig. 1), as lower scores indicated better performance. The performance ceiling was reached after 23 administrations of the TMT-A and change over time explained 56% of the variability in TMT-A scores within players: (5.46–2.41)/5.46 = 0.56 (see Tables 1 and 2). Performance improved by 23.6% from T1-T22 to the ceiling (T23-T57) and improved by 47.2% from first administration (T1) to the ceiling.

**Table 2:** Fit of models of TMT-A scores with outliers excluded

	Model 1	Model 2	Model 3	Model 4	Model 5	Model 6
Fixed Effect	Coefficient (*SE*)	Coefficient (*SE*)	Coefficient (*SE*)	Coefficient (*SE*)	Coefficient (*SE*)	Coefficient (*SE*)
TMT-A	10.41 (0.31)^*^	15.94 (0.27)^*^	16.24 (0.59)^*^	16.41 (0.63)^*^	17.49 (0.95)^*^	17.49 (0.92)^*^
Time		0.97 (0.02)^*^	1.03 (0.09)^*^	1.03 (0.09)^*^	1.21 (0.15)^*^	1.21 (0.15)^*^
Pre-season				−0.16 (0.27)	−0.83 (0.79)	−0.74 (0.77)
Match day				−0.01 (0.18)	−5.70 (2.26)^*^	−5.21 (2.11)^*^
≤7 days of match				−0.21 (0.15)	−1.46 (0.79)	−1.51 (0.72)^*^
>7 days of match				—	—	—
Time^*^ Pre-season					0.30 (0.24)	0.33 (0.23)
Time^*^ Match Day					−0.86 (0.34)^*^	−0.78 (0.31)^*^
Time^*^ ≤7 days of match					−0.21 (0.13)	−0.22 (0.12)
Time^*^ >7 days of match					—	—
**Random Effect**	**Parameter (*SE*)**	**Parameter (*SE*)**	**Parameter (*SE*)**	**Parameter (*SE*)**	**Parameter (*SE*)**	**Parameter (*SE*)**
*Level 2 variance (between):*						
Intercept variance	3.12 (0.96)^*^	1.75 (0.48)^*^	22.62 (6.27)^*^	22.59 (6.26)^*^	22.88 (6.32)^*^	22.45 (6.48)^*^
Slope variance			0.25 (0.07)^*^	0.25 (0.07)^*^	0.25 (0.07)^*^	0.25 (0.07)^*^
Intercept-slope covariance			−2.29 (0.64)^*^	−2.29 (0.64)^*^	−2.32 (0.64)^*^	−2.27 (0.66)^*^
*Level 1 variance (within):*						
Residual variance	5.46 (0.22)^*^	2.41 (0.10)^*^	1.79 (0.07)^*^	1.79 (0.07)^*^	1.78 (0.07)^*^	1.88 (0.09)^*^
Deviance (−2LL)	5738	4734	4458	4459	4450	4372

*Note*: *SE* = standard error; ^*^P < 0.05; — redundant as >7 days of match is the reference category; −2LL = −2 restricted log likelihood.

**Figure 1:**
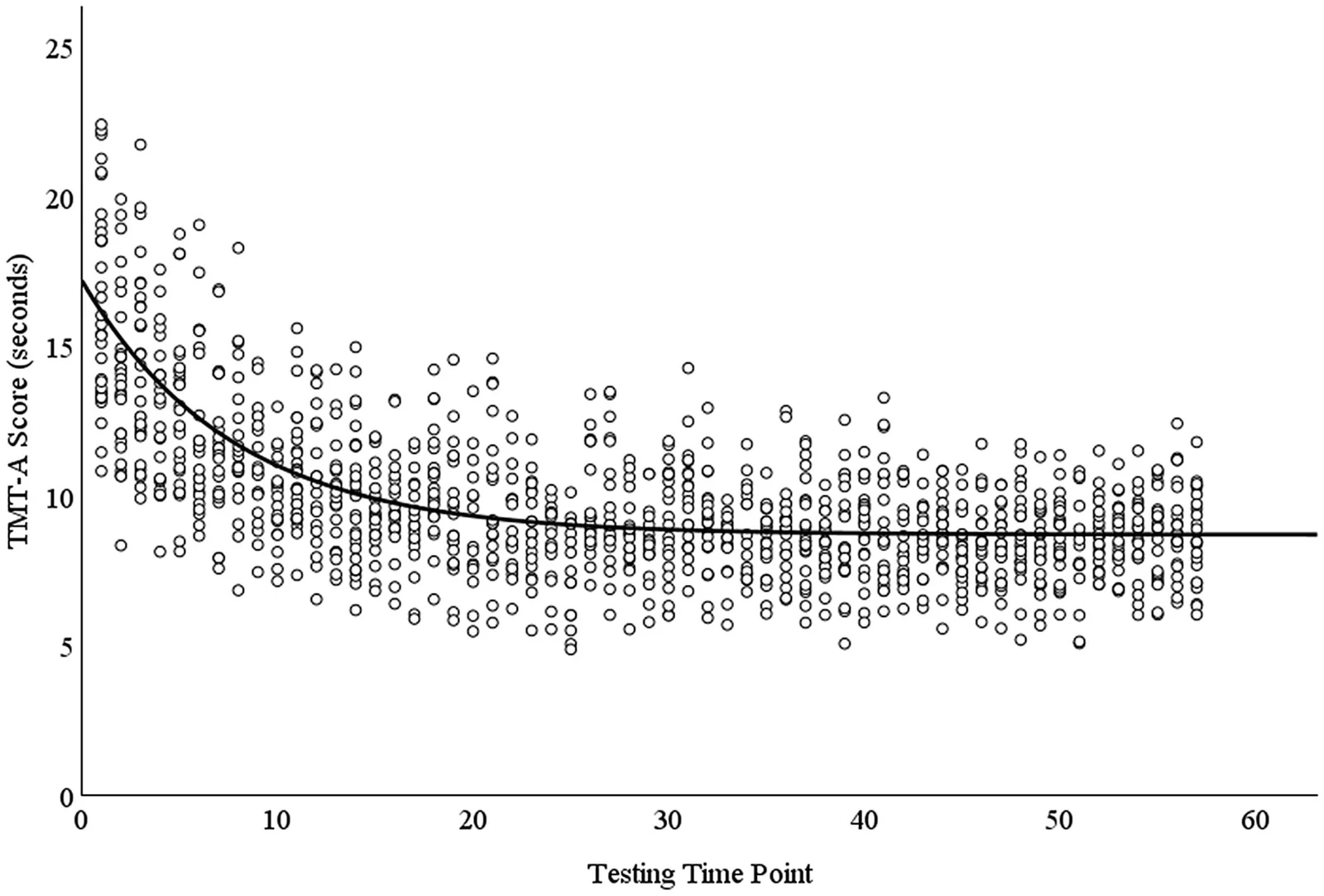
TMT-A Scores across Testing Time-Points*.*

The random slope model (Model 3) found that there was a substantial improvement in model fit by allowing the intercepts to vary by time. The -2LL value further reduced and a chi-square distribution table indicated that this was a significant decrease (*P* < 0.001) and an improvement in the model fit. As can be seen in Table 2, allowing the slopes to vary by time increased the intercept variance, which indicated that there were significant differences between player differences in TMT-A scores at first testing. The random slope indicated that there was a significantly different effect of time on TMT-A scores between the players. Although the slope variance was significant (*P* < 0.001), the parameter estimate (0.25) indicated a slight fanning out of individual slopes. The covariance between the intercept and slope was also significant (*P* < 0.001) and negative (−2.29), which indicated that players who had decreased TMT-A scores over time were more likely to have had higher TMT-A scores at first testing. The inclusion of a random effect of time also reduced the within-individual variance. The additional variance explained was 11% [(2.41–1.79)/5.46 = 0.11; see Table 2] and indicated that much of the improvement in performance on the TMT-A within each player was due to test repetition.

The inclusion of testing day as a fixed effect and as an interaction term had a negligible effect on level 1 and 2 variances (see Models 4 and 5, Table 2). These results indicated that testing day did not have a significant effect on TMT-A scores and the only significant interaction term was when the testing day was on a match day. The estimate of the slope was −0.86 (*SE* = 0.34) and indicated that match day TMT-A scores increased over time as exponential decay modelled a decrease in scores over time. The inclusion of modelling the non-independence of TMT-A performance significantly improved the model fit (reduction in -2LL points, *P* < 0.001; see Model 6, Table 2). There was a significant autoregression correlation (*ρ* = 0.33, *P* < 0.001), which indicated a positive relationship between players’ TMT-A scores at adjacent time-points. This finding is important; previous test experience contributes to performance and taken with the amount of variance explained by time, indicates a learning effect with test repetition.

### TMT-B

The unconditional model for the TMT-B found that there was variability in the intercepts between the players (Model 1; see Table 3). As can be seen in Table 3, the intercept variance was significantly greater than zero (*P* = 0.001). The ICC(1) found that 26% of the variability in TMT-B scores was due to differences between players [19.58/(55.58 + 19.58) = 0.26; see Table 3]. When time was added as a fixed effect (Model 2), the model fit improved as indicated by a decrease in deviance points (see Table 3). The positive effect of the exponential decay model of time (*β* = 0.97, *SE* = 0.2, *P* < 0.001) indicated that TMT-B scores decreased over time and then stabilized after reaching a ceiling (see Table 1 and Fig. 2). The plateau was also reached after 23 administrations of the TMT-B, and a large proportion of the variability (64%) within players was explained by time [(55.58–19.87)/55.58 = 0.64; see Tables 1 and 3]. Performance improved by 38.9% from T1-T22 to the ceiling and by 65.6% from first administration (T1) to the ceiling.

**Table 3:** Fit of models of TMT-B scores with outliers excluded

	Model 1	Model 2	Model 3	Model 4	Model 5	Model 6
Fixed Effect	Coefficient (*SE*)	Coefficient (*SE*)	Coefficient (*SE*)	Coefficient (*SE*)	Coefficient (*SE*)	Coefficient (*SE*)
TMT-B	19.10 (0.80)^*^	38.83 (0.72)^*^	39.52 (2.02)^*^	39.57 (2.12)^*^	40.66 (3.08)^*^	40.74 (3.00)^*^
Time		0.97 (0.02)^*^	1.01 (0.08)^*^	0.99 (0.09)^*^	1.04 (0.14)^*^	1.06 (0.13)^*^
Pre-season				−0.03 (0.74)	0.71 (2.41)	0.71 (2.34)
Match day				−0.86 (0.49)	−2.56 (7.78)	−7.88 (7.30)
≤7 days of match				−0.38 (0.40)	−2.25 (2.43)	−2.45 (2.24)
>7 days of match				—	—	—
Time^*^ Pre-season					0.51 (0.18)^*^	0.48 (0.17)^*^
Time^*^ Match Day					−0.08 (0.33)	−0.32 (0.31)
Time^*^ ≤7 days of match					−0.09 (0.11)	−0.11 (0.11)
Time^*^ >7 days of match					—	—
**Random Effect**	**Parameter (*SE*)**	**Parameter (*SE*)**	**Parameter (*SE*)**	**Parameter (*SE*)**	**Parameter (*SE*)**	**Parameter (*SE*)**
*Level 2 variance (between):*						
Intercept variance	19.58 (6.10)^*^	11.12 (3.09)^*^	51.82 (15.26)^*^	51.71 (15.23)^*^	54.28 (16.08)^*^	50.13 (15.40)^*^
Slope variance			0.22 (0.06)^*^	0.22 (0.06)^*^	0.22 (0.06)^*^	0.22 (0.06)^*^
Intercept-slope covariance			−3.07 (0.90)^*^	−3.07 (0.90)^*^	−3.19 (0.94)^*^	−3.04 (0.92)^*^
*Level 1 variance (within):*						
Residual variance	55.58 (2.28)^*^	19.87 (0.81)^*^	12.66 (0.53)^*^	12.66 (0.53)^*^	12.49 (0.52)^*^	13.42 (0.68)^*^
Deviance (−2LL)	8536	7287	6843	6839	6826	6754

*Note. SE* = standard error; ^*^P < 0.05; — redundant as >7 days of match is the reference category; −2LL = −2 restricted log likelihood.

**Figure 2:**
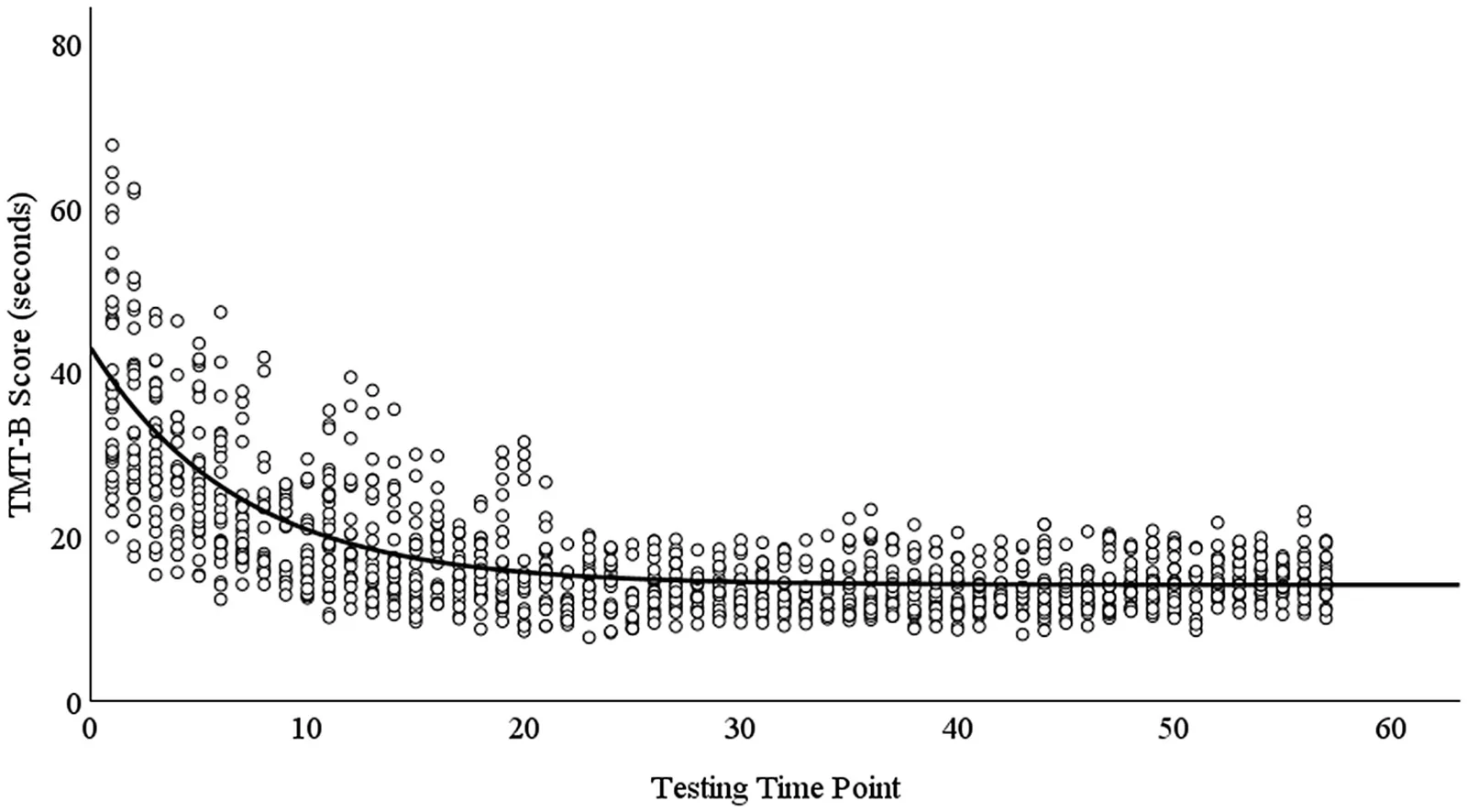
TMT-B Scores across Testing Time-Points*.*

Model fit was improved further by allowing the intercepts to vary by time (Model 3) as indicated by a statistically significant reduction in deviance points (*P* < 0.001). As can be seen in Table 3, allowing the slopes to vary by time increased the intercept variance, which indicated that there were significant between player differences in TMT-B scores at first testing. The slope variance was also significant and indicated a slight fanning out of individual slopes. The intercept-slope covariance indicated that players who had decreased TMT-B scores over time were more likely to have had higher TMT-B scores at first testing. The addition of a random effect of time further reduced the within-participant variance by 12% [(19.58–12.66)/55.58 = 0.12; see Table 3]. Similar to the TMT-A findings, test repetition explained much of the improvement in performance within players.

The inclusion of testing time-point had negligible effects on the residual and intercept variances and like the TMT-A analysis, indicated that testing time-point did not influence TMT-B scores (Model 4). The only significant interaction term was when the testing day was pre-season (Model 5; see Table 3). The slope coefficient indicated that pre-season TMT-B scores significantly decreased over time. This finding was unsurprising, given the rapid improvement in TMT-B performance during this period of time (T1–T3; see Fig. 2). Modelling the non-independence of TMT-B performance significantly improved the model fit (reduction in -2LL points, *P* < 0.001; see Model 6, Table 3). Again, there was a significant positive relationship between the players’ TMT-B scores at adjacent time-points (*ρ* = 0.34, *P* < 0.001). This result, along with the amount of variance explained by time, indicated that there was also a learning effect with test repetition on the TMT-B.

## Discussion

The aim of the study was to explore patterns of inter- and intra-individual performance on the TMT-A and TMT-B with high frequency testing over the course of a playing season in elite rugby league players without concussion. Time was the greatest predictor of both TMT-A and TMT-B scores; test performance improved until it reached a plateau and then remained stable. Importantly, the inclusion of a random effect of time facilitated examination of inter- and intra-individual test performance. There was significant between-player variability at first testing for both TMT-A and TMT-B, and performance significantly differed over time between the players for both tests. Specifically, players who had decreased test scores over time were more likely to have had higher scores at first testing. The reduction of within-individual variance indicated that improvement in TMT-A and TMT-B performance within each player was due to test repetition. This latter conclusion was further supported by the improvement in model fit when within-person variance was accounted for and indicated a significant relationship between the players’ test scores at adjacent time-points. Lastly, inter- and intra-individual performance on the TMT-A and TMT-B was not significantly influenced by testing time-point (e.g., pre-season, match day, post-match day). In summary, performance on the TMT-A and TMT-B improved over time until it reached a ceiling after which it was stable. Importantly, performance was significantly different between players at first testing and over the course of a playing season which indicated performance heterogeneity within one cohort of elite rugby league players.

The findings of the current study agree with previous studies that have found practice effects with the TMT in healthy adult and athlete populations (e.g., Bartels et al., 2010; Collie et al., 2006; Dikmen et al., 1999; Julien et al., 2023; Levine et al., 2004). The current study contributes to the literature as few studies have conducted high frequency serial assessment over 8 months. The overall pattern of TMT performance is similar to that of healthy adults (Bartels et al., 2010) in that performance improves over time until it reaches a ceiling and then plateaus. This pattern was also found in semi-professional rugby union athletes with the King-Devick test administered over a 21 time-point period (assessments were at least 7 days apart) during elective training sessions (Gunasekaran et al., 2020). Evidence of variability in TMT performance at first testing in the current study is in line with Fuller and coworkers (2021) who found a wide range of TMT-B scores at baseline (75.7 s). A unique contribution to the literature of the current study is the reporting of random effects models which indicated different patterns of TMT performance between players. Whilst these findings could be interpreted to argue against use of the TMT to monitor cognitive performance in athletes at risk of concussion, mapping inter- and intra-individual patterns of behaviour contribute to further understanding test performance. A consideration of methods to address variable test performance and practice effects is warranted in future studies.

Significant improvements in performance over time call into question the reliability of baseline assessment in athletes at risk of concussion. An optimal approach is to conduct high frequency test administration until the performance ceiling is reached. The ceiling value can then be used as a measure of baseline performance. In the current study, the plateau for both TMT-A and TMT-B was 23 administrations. Gunasekaran and coworkers (2020) identified that 30 administrations of the King-Devick test were needed to reach performance ceiling. However, these numbers of test administrations are prohibitive in practice in athletic settings. Gunasekaran and coworkers (2020) concluded that regular testing over a playing session with at least six administrations could contribute to a more reliable baseline measure of performance, which aligns with the high frequency testing schedule used in Bartels and coworkers (2010). One approach to identify and address variable TMT performance at first testing is to use percentile ranks (cf. Fuller et al., 2021) and calculate the B/A ratio. The use of percentile ranks, the B/A ratio and regular testing can address the challenges presented by variable TMT performance and facilitate more reliable measurement of pre-injury test performance.

### Study limitations

Whilst the current study has made a unique contribution to understanding patterns of TMT performance in elite rugby league players without concussion, the study does have limitations. Data were only collected from one team and as such have limited generalizability. Previous studies in rugby players have included larger samples from multiple clubs and countries (Fuller et al., 2021; Julien et al., 2023), enhancing the representativeness of their findings. Education level and learning differences were not recorded in the current study. Higher level of education was linked with better TMT-B performance (Julien et al., 2023) and previous normative data studies have accounted for education and IQ (e.g., Hays, 1995; Stanton et al., 1984; Tombaugh, 2004). There is the potential for dyslexia, dyscalculia, and attention deficit hyperactivity disorder (ADHD) to influence executive functions, such as those measured by the TMT (e.g., Hale et al., 2009; Lonergan et al., 2019; Närhi et al., 1997; Szucs et al., 2013). However, learning differences are rarely considered in studies investigating cognitive functioning in rugby players and the effects of such conditions on test performance are not known. The playing position was also not recorded. This is an important consideration as TMT-B performance differed between forwards and backs, suggesting that different cognitive profiles in players are due to specific position-related skills (Julien et al., 2023). The shorter version of the TMT used in the current study restricts comparability with previous studies using the original sized version. However, there is increasing use of shorter versions of the TMT (e.g., Fuller et al., 2021) and this study extends current understanding of TMT performance on abridged versions as serial assessment was conducted over one playing season.

## Conclusion

The current study identified patterns of practice effects both between and within-players. These data are important as there has been little investigation of cognitive functioning in rugby players over the course of one playing season. Practice effects are a common feature of neuropsychological assessment (see Calamia et al., 2012) and several methods are advocated for reducing practice effects. For example, multiple baseline measurements (e.g., Richardson et al., 2017), calculation of the reliable change index (Goldberg et al., 2015), and use of alternate versions of the TMT (e.g., Atkinson et al., 2011; Beglinger et al., 2005; Wagner et al., 2011). The current study has contributed to further understanding pre-injury cognitive performance in elite rugby players and indicates the need for future studies to implement strategies for reducing practice effects. Most importantly, these findings are relevant when assessing cognitive functioning in athletes at risk of concussion. This study emphasizes the need to account for variability in performance pre-injury, for example, by using percentile ranks and administering tests multiple times. Furthermore, we recommend assessing potential performance confounds (e.g., learning differences) and the use of methods to reduce practice effects, such as using alternative versions of tests.

## Supplementary Material

Supplementary_materials_acag082
