# Supplementary material for: The effects of serial assessment using the trail making test in elite rugby league players
Source: Arch Clin Neuropsychol. 2026 Oct 15;41(7):acag082. doi: 10.1093/arclin/acag082 (PMC13671231; doi:10.1093/arclin/acag082)
Supplement: Supplementary file 1 [file supplementary_materials_acag082.docx]

**Supplementary Analysis: TMT B/A Ratio**

The models detailed in the Statistical Analysis were fitted to the TMT B/A ratio data. Curve fitting of change over time found that a quadratic model provided the best fit for these data *R^2^* = .20 (see Figure S1). The initial unconditional model (Model 1) estimated within and between-individual variances. The quadratic model of time was added as a fixed effect in Model 2 and as a random effect in Model 3. Models 4 and 5 tested whether testing time point (e.g., pre-season, match day) and the quadratic model of time*testing time-point interaction influenced TMT B/A ratios. Within-person variance was modelled in Model 6 to test for serial correlation of ratios between adjacent time-points.

**Figure S1.**

*TMT B/A Ratios across Testing Time-Points.*

Alt-Text: Figure S1 displays all the TMT B/A ratios from T1 to T57. Time is on the x-axis and TMT B/A ratios are on the y-axis. A quadratic curve is fitted to the data.

The initial unconditional model (Model 1) found that the variance term for the intercept was significantly greater than zero (see Table S1), which indicated that there was significant variability in the intercepts between the players. The ICC(1) was calculated: 0.08/(0.20 + 0.08) = 0.29 and indicated that 29% of the variance in TMT B/A ratios was attributable to differences between players. The inclusion of the fixed effect of time in Model 2 improved the model fit. The coefficient indicated that there was a positive relationship between time and TMT B/A ratios; therefore, ratios decreased over time followed by an increase in the latter testing time points (see Figure S1). Change over time explained 20% of the variability in TMT B/A ratios within players: (0.20 – 0.16)/0.20 = 0.20 (see Table S1). The vertex coordinates were calculated; the vertex was at testing time-point 23.5 with a ratio score of 1.81. This is a 27.2% reduction from first TMT administration (T1).

The random slope model (Model 3) found that there was a substantial improvement in model fit by allowing the intercepts to vary by time. The -2LL value further reduced and a chi-square distribution table indicated that this was a significant decrease (*p* < .001). As can be seen in Table S1, allowing the slopes to vary by time increased the intercept variance, which indicated that there were significant differences between player differences in TMT B/A ratios at first testing. The random slope indicated that there was a significantly different effect of time on TMT B/A ratios between the players. Although the slope variance was significant (*p* < .001), the parameter estimate (0.65) indicated a slight fanning out of individual slopes. The covariance between the intercept and slope was also significant (*p* < .001) and negative (-1.11), which indicated that players who had decreased TMT B/A ratios over time were more likely to have had higher ratios at first testing. The inclusion of a random effect of time also reduced the within-individual variance. The additional variance explained was 15% [(0.16 – 0.13)/0.20 = 0.15; see Table S1] and indicated that a proportion of the change in ratios within each player was due to test repetition.

The inclusion of testing day as a fixed effect and as an interaction term had a negligible effect on within and between player variances (see Models 4 and 5, Table S1). The results indicated that pre-season testing (T1-T3) had an effect on TMT B/A ratios and the time*pre-season interaction coefficient indicated that the ratios significantly decreased during this period of time and is likely due to the improvement in TMT-B scores during this time. The inclusion of modelling the non-independence of TMT-A performance did not change the model fit (see -2LL value Model 6, Table S1). The autoregression correlation was not significant (*p* < .445) and indicated that the ratios at adjacent time-points were not related.

In summary, allowing the intercepts to vary by time provided further evidence of significant differences between players and emphasises the need to account for inter-individual variability at first testing and over time. Lamberty et al. (1994) proposed a TMT B/A ration cut-off of ≥ 3.0 to indicate impairment and whilst this was questioned by Drane et al. (2002), it is a useful marker to identify individuals who may have very quick TMT-A completion times and/or very slow TMT-B completion times.

**Table S1.**

*Fit of Models of TMT Ratios.*

|  | Model 1 | Model 2 | Model 3 | Model 4 | Model 5 | Model 6 |
| --- | --- | --- | --- | --- | --- | --- |
| Fixed Effect | Coefficient (*SE*) | Coefficient (*SE*) | Coefficient (*SE*) | Coefficient (*SE*) | Coefficient (*SE*) | Coefficient (*SE*) |
| TMT B/A Ratio | 1.83 (0.05) * | 2.32 (0.05) * | 2.35 (0.10) * | 2.23 (0.11) * | 2.07 (0.15) * | 2.07 (0.15) * |
| Time |  | 0.95 (0.05) * | 1.03 (0.15) * | 0.89 (0.15) * | 0.57 (0.25) * | 0.57 (0.25) * |
| Pre-season |  |  |  | 0.19 (0.06) * | 0.45 (0.13) * | 0.45 (0.13) * |
| Match day |  |  |  | -0.03 (0.05) | 0.23 (0.27) | 0.23 (0.27) |
| ≤ 7 days of match |  |  |  | 0.04 (0.04) | 0.21 (0.12) | 0.21 (0.12) |
| > 7 days of match |  |  |  | -- | -- | -- |
| Time * Pre-season |  |  |  |  | 2.52 (1.08) * | 2.51 (1.08) * |
| Time * Match Day |  |  |  |  | 0.45 (0.40) | 0.45 (0.40) |
| Time * ≤ 7 days of match |  |  |  |  | 0.33 (0.22) | 0.33 (0.22) |
| Time * > 7 days of match |  |  |  |  | -- | -- |
|  |  |  |  |  |  |  |
| Random Effect | Parameter (*SE*) | Parameter (*SE*) | Parameter (*SE*) | Parameter (*SE*) | Parameter (*SE)* | Parameter (*SE*) |
| *Level 2 variance (between):* |  |  |  |  |  |  |
| Intercept variance | 0.08 (0.02) * | 0.06 (0.02) * | 1.94 (0.57) * | 1.91 (0.56) * | 1.92 (0.56) * | 1.91 (0.56) * |
| Slope variance |  |  | 0.65 (0.19) * | 0.64 (0.18) * | 0.65 (0.18) * | 0.64 (0.18) * |
| Intercept-slope covariance |  |  | -1.11 (0.32) * | -1.10 (0.32) * | -1.10 (0.32) * | -1.09 (0.32) * |
| *Level 1 variance (within):* |  |  |  |  |  |  |
| Residual variance | 0.20 (0.01) * | 0.16 (0.01) * | 0.13 (0.01) * | 0.13 (0.01) * | 0.13 (0.01) * | 0.13 (0.01) * |
|  |  |  |  |  |  |  |
| Deviance (-2LL) | 1580 | 1300 | 1142 | 1143 | 1135 | 1135 |

*SE* = standard error; * *p* < .05; -- redundant as > 7 days of match is the reference category; -2LL = -2 restricted log likelihood.
